# The cost of a disease targeted for elimination in Brazil: the case of schistosomiasis mansoni

**DOI:** 10.1590/0074-02760180347

**Published:** 2019-01-14

**Authors:** Gilmara Lima Nascimento, Helio Milani Pegado, Ana Lúcia Coutinho Domingues, Ricardo Arraes de Alencar Ximenes, Alexander Itria, Luciane Nascimento Cruz, Maria Regina Fernandes de Oliveira

**Affiliations:** 1 Universidade de Brasília, Núcleo de Medicina Tropical, Brasília, DF, Brasil; 2 Universidade Federal de Pernambuco, Recife, PE, Brasil; 3 Universidade Federal de Goiás, Goiânia, GO, Brasil; 4 Instituto de Avaliação de Tecnologias em Saúde, Porto Alegre, RS, Brasil

**Keywords:** schistosomiasis mansoni, cost of illness

## Abstract

**BACKGROUND:**

Schistosomiasis mansoni is a poverty-related parasitic infection that has a variety of clinical manifestations. We consider the disability and deaths caused by schistosomiasis unacceptable for a tool-ready disease. Its condition in Brazil warrants an analysis that will enable better understanding of the local health losses and contribute to the complex decision-making process.

**OBJECTIVE:**

This study estimates the cost of schistosomiasis in Brazil in 2015.

**METHODS:**

We conducted a cost of illness study of schistosomiasis mansoni in Brazil in 2015 based on a prevalence approach and from a societal perspective. The study included 26,499 schistosomiasis carriers, 397 hepatosplenic cases, 48 cases with the neurological form, 284 hospitalisations, and 11,368.26 years of life lost (YLL) of which 5,187 years are attributable to economically active age groups.

**RESULTS:**

The total cost of schistosomiasis mansoni in Brazil was estimated to be US$ 41,7million in 2015 with 94.61% of this being indirect costs.

**CONCLUSIONS:**

The economic burden of schistosomiasis mansoni in Brazil is high and results in the loss of productivity. Its persistence in Brazil is a challenge to public health and requires inter-sectorial interventions in areas such as indoor water supply, basic sanitation, and education.

Schistosomiasis mansoni is a poverty-related parasitic infection characterised by various clinical manifestations: milder symptoms such as malnutrition, anaemia, abdominal pain, and diarrhoea; and more severe conditions including temporary paralysis (neurological cases) and upper gastrointestinal bleeding (hepatosplenic cases).^1^ Portal fibroses may reach up to 20% of infected cases in endemic areas.^2^ It is transmitted mainly through skin contact with fresh water contaminated by *Schistosoma mansoni* larvae. In endemic areas with poor sanitation, human faeces containing *S. mansoni* eggs may reach water sources through snail hosts, completing the transmission cycle.^3^ Transmission, infection, morbidity, and mortality are closely related to social, economic, and environmental conditions.^4^

Historically, schistosomiasis mansoni has been endemic in Brazil. However, its geographical distribution in Brazilian states is not homogenous, even in endemic cities. The country has 27 states, and schistosomiasis is present in 19 of these states where in nine states the disease is endemic. This disease has focal transmission in 10 states.^5^ For more than 20 years, schistosomiasis has been the target of a National Control Program (PCE) aimed at the detection and treatment of infected people. Throughout these years, the prevalence, morbidity, and mortality related to schistosomiasis have decreased.^6^ ^,^ ^7^ However, severe cases and death continue to occur. Schistosomiasis is considered a tool-ready disease, and Brazil is a country that theoretically has all the necessary conditions to eliminate chronic and severe cases of schistosomiasis.

According to official health information systems, there were 175 hospitalisations and 461 deaths due to schistosomiasis in 2015.^7^ ^,^ ^8^ Considering the natural history of schistosomiasis, the common chain of events involved in death by schistosomiasis mansoni^1^ ^,^ ^3^ and the social condition of the cases, people at the late stages of the disease would require hospitalisation, normally in public health facilities. Therefore, the number of hospitalisations appears to be underestimated.

As far as surveillance is concerned, two information systems are available in Brazil. SINAN_ESQ, a passive system, is exclusively aimed at severe cases and new cases found in areas where schistosomiasis transmission has not been previously reported. The other system, SISPCE, applies to known endemic areas and focuses on active case searches accomplished through community stool surveys. In 2014, more than 6,500 cases were reported to SINAN_ESQ and 33,357 carriers of schistosomiasis mansoni were declared in SISPCE.^9^ ^,^ ^10^

Brazil has a complicated epidemiological scenario especially in many schistosomiasis endemic states and municipalities characterised by the prevalence of chronic diseases such as hypertension and diabetes, incidence of infectious diseases, seasonal dengue epidemics, and most recently, Zika virus infection with the emergence of congenital neurological damages. Nevertheless, the persistent disabilities and deaths caused by schistosomiasis (which consequently increase health expenditures) remain to be addressed. This warrants an analysis that would enhance the understanding of health-related losses and contribute to the complex decision-making process.

In studying the costs associated with a disease, an important hypothesis is that these costs would otherwise provide potential benefits for the population if such a disease is either controlled or eliminated. Thus, quantifying the opportunity costs is greatly relevant for the affected population and poverty-related diseases.^11^ Therefore, this study estimates the cost of schistosomiasis in Brazil in 2015.

## MATERIALS AND METHODS

Based on the prevalence approach, we conducted a study on the cost of the disease caused by *S. mansoni* ^12^ in Brazil to determine the economic burden of schistosomiasis in 2015. We used the Brazilian Unified Health System (SUS) and employed a societal perspective with the Brazilian population living with schistosomiasis as the target.

*Definition of the study population* - In this study, the population used to estimate the cost burden of the disease was based on the records of individuals who tested positive for *S. mansoni* reported to SISPCE, those with neurological and hepatosplenic forms of the disease as registered in SINAN_ESQ, those who were hospitalised due to schistosomiasis as registered in the Hospital Information System (SIH-SUS) and those who died due to schistosomiasis as reported in the Mortality Information System (SIM). This population is identified as Base Case (Table I).

**TABLE I:** Detailed study population according to the clinical form and events considered for disease cost estimations. Brazil, 2015

Category of cases/ events for cost estimation	Base case	Scenario A	Scenario B	Scenario C
Performed stool sample tests	654,321	449,791	2,024,061	3,639,222
*Schistosoma mansoni* infection carriers	26,499	449,791	2,024,061	3,639,222
Hepatosplenic form of the disease (2.5% of infected people)	397	11,245	50,601	90,981
Neurologic form of the disease (0.7% of infected people)	48	3,149	14,168	25,475
Hospitalisations (6.4% of chronic forms)	275	921	4,145	7,453
Hospitalisations in intensive therapy care unit (3.2% of hospitalisations)	9	29	133	239
Cases that paid transportation for treatment (22.5% of severe forms)	100	3,238	14,573	26,202
Users that needed to pay a caregiver (3.5% of severe forms)	16	504	2,226	4,076
Economically active cases for which work leave and disease aid costs are applied among neurological forms (22%)	87	2,473	11,132	20,016
Economically active cases for which work leave and disease aid costs are applied among neurological forms (22%)	10	693	3,117	5,604
Years of life lost due to premature death (constant)	5,187	5,187	5,187	5,187

Base case: records from national information systems are the source of the numbers regarding the categories of cases/events. Measurements were applied only for cases with demand for transportation, caregiver, leave, and disease aid; scenario A: cases estimated based on the confidence interval lower limit (95%CILL) of the prevalence estimated in the national survey of schistosomiasis; scenario B: cases estimated based on the prevalence estimated in the national survey of schistosomiasis; scenario C: cases estimated based on the 95%CI upper limit of the prevalence estimated in the national survey of schistosomiasis.

The populations simulated for the sensitivity analysis considered the results from the National Survey on Schistosomiasis and Soil-transmitted helminths,^5^ using estimate and confidence interval limits (95% CI). In the survey, the prevalence of infection was estimated to be 0.99% (IC 95% 0.22% - 1.78%). The distribution of infected people with clinical forms included in this study was based on the registration of cases from the state of Minas Gerais (SINAN_ESQ_MG) as reported at SINAN_ESQ, considering that Minas Gerais established that any clinical form of schistosomiasis is a disease of compulsory notification. The SINAN_ESQ_MG provided reports of the positive cases (whether symptomatic or not), which were identified in active searches or local surveys, as well as late or ectopic forms identified in a passive manner. In the other states of the Federation, reports to SINAN_ESQ are restricted to severe, ectopic, or positive cases. We considered reports from the SINAN_ESQ_MG as representative of the disease distribution in terms of clinical form given that SISPCE only registers the infection, not distinguishing the clinical form of the disease, which is essential information to estimate costs.

We found 6,305 registered cases from the national SINAN_ESQ database, of which 5,860 (92.9%) were intestinal or hepatointestinal, 397 (6.3%) were hepatosplenic, and 48 (0.7%) were neurological cases. After analysing the SINAN_ESQ_MG database, the cases were stratified according to the clinical forms established in the schistosomiasis investigation form. We observed 3,708 cases (91; 2.5%) hepatosplenic, 26 (0.7%) neurological, and 3,591 (96.8%) intestinal or hepatointestinal cases from the SINAN_ESQ_MG database. Such proportions were applied to the number of estimated infected individuals using the prevalence and the 95% CI to simulate the three scenarios of the sensitivity analyses. These populations were identified as Scenario A (lower limit of the 95% CI), Scenario B (estimate), and Scenario C (upper limit of the 95% CI). Table I provides a description of the number of cases and events considered for estimating the cost of schistosomiasis.

The potential years of life lost (YLL) by premature death was used to estimate part of the indirect costs. YLL was calculated using records of deaths caused by schistosomiasis from the SIM. Relevant information from the death certificate (DC) signifying the cause of death, were used as the main selection criteria. Such database showed 529 deaths, and schistosomiasis was not indicated as the basic cause in 155 deaths. We conducted an individual assessment of each record to identify deaths that indicated schistosomiasis as the basic cause, but among the 155 records that had CID10 of schistosomiasis in part 1 of the DC, we could not define it as the basic cause. We attempted to develop a clinical causal relationship between the CID completed in lines ‘a’ to ‘d’ from the DC and to identify the basic cause of death according to the sequence of DC completion. We considered the causes of deaths due to schistosomiasis to include cases of hematemesis, bleeding of oesophageal varices, hypovolemic shock by hematemesis, death due to hepatic insufficiency, and portal hypertension with portal vein thrombosis, encompassing 406 deaths.

YLL was estimated based on the product between the number of deaths and the life expectancy at the age of death. Records on 406 deaths were stratified according to gender and detailed age range (every five years from 0 to 65 years or older: 0, 1-4, 5-9, 10-14, and so on up to 65). The median of ages from each age range was established as the mean age in which there was occurrence of death in the respective age group. The study used the life table from the ‘Brazilian Institute of Geography and Statistics of 2015’ with life expectancies of 71.9 and 79.1 years for males and females, respectively.

*Categories, costs and hypotheses* - We made an extensive cost inventory for the ‘direct healthcare costs’ category including the items related to stool sample investigations or search for cases in the community; the methods used for diagnosing the disease’s clinical forms and following-up the patients’ evolution; and the infection treatments and clinical repercussions of chronic and neurological forms. Transportation for treatment and caregiver’s payment were considered non-healthcare direct costs. These costs were identified through interviews with 174 schistosomiasis patients that had outpatient follow-up visits (carried out from July to December of 2015) at the Hospital das Clínicas from Universidade Federal de Pernambuco (AE/HC/UFPE). Indirect costs included values attributed to YLL due to premature death, work leave, and disease aid of the economically active population and were estimated using the human capital method. This method estimates present human capital value as a representation of a person’s future gains based on the hypothesis that the person’s future gains represent his future productivity.^12^

To establish the cost items included in this study as well as their measurement and valuation parameters (see Table II), we used specific schistosomiasis surveillance guides from the Brazilian Ministry of Health. We also used information from specialists in the basic protocol of patients with digestive forms of the disease, and standard laboratorial operational procedures and data collected in the outpatient clinic of schistosomiasis (AE/HC/UFPE) as references. The amounts attributed to the items were based on operational and epidemiological parameters obtained from the analysis of SISPCE, SINAN_ESQ, SIH-SUS, and clinical protocols. When a certain parameter is unavailable, we estimated the values using clinical recommendations as reference.

The monetary values attributed to the items were searched in systems or tables of prices within a domestic scope. If an item’s price varied throughout the year, we used a monthly average. When we could not find a single national reference, we chose the price reference from endemic states. The parameters described in text box one were applied to the values with details provided in the Supplementary data (Table). The years of life lost due to premature death remained constant in all the scenarios. We estimated the values for each investigated case included in the survey and for each hepatosplenic schistosomiasis and neurological case. Based on the individual values, we estimated the cost of the disease for the base case and performed a sensitivity analysis in three different scenarios. The values obtained in Brazilian Reais were converted to US dollars based on the prevailing exchange rate on July 1,as quoted from 2015 (3.263).

We used the following data sources: SIM; SIH-SUS; medical records and interviews with patients from AE/HC/UFPE; scientific literature; management system of the procedures, drugs and orthesis, prosthesis, and special devices from the Brazilian Unified Health System (SIGTAP); price database of the Brazilian Department of Health; websites from the Planning Department, Health Department, Health State and City Offices; Public Tender Offers; and clinical protocols.

## RESULTS

The study’s Base Case included costs for 26,499 schistosomiasis carriers (infected people), 20,646 (77.9%) positive case records from the SISPCE, and 5,853 (22.1%) reported cases in SINAN. Regarding severe forms of the disease and measured events in the costs, we considered 397 hepatosplenic cases (SINAN), 48 cases with the neurological form (SINAN), 275 hospitalisations in wards (SISUS), and nine hospitalisations in ICUs (SISUS).

We included 406 deaths, of which 269 (51.0%) were males and 127 (31.2%) occurred in ages that are considered economically active. Pernambuco and Minas Gerais were the residence states of 46.5% (189) of the deaths. The states with the highest standardised mortality rate caused by schistosomiasis were: Alagoas (1.48/100,000 residents), Pernambuco (1.20/100,000 residents), and Sergipe (0.61/100,000 residents) (Table III).

**TABLE II:** Assumptions applied to estimate the cost of *Schistosoma mansoni*. Brazil, 2015

Category of costs	Type of cost	General hypotheses
Direct health care costs	Stool sample investigations in the endemic or focal cities	The stool sample survey is the action to establish the treatment strategy based on the percentage of positivity in the place where the transmission has been established, with the aim of decreasing the prevalence and avoiding the evolution to chronic and severe forms of the disease. Considered items: community’s action to distribute the specimen containers by a health community worker, sample collection, laboratorial sample processing and a Kato-katz thick smear by a technician in clinical analysis, result delivery, treatment administration during delivery and registration in specific form.
	Diagnosis of the chronic digestive and neurological forms	Imaging exams and laboratorial analyses recommended in guides on disease clinical management or specialised service routines. The proportion of cases, according to each clinical form, which used imaging exams, was measured from the information obtained in the Brazilian Information System for Notifiable Diseases.
	Hospitalisations	The total of hospitalisations for the Base Case was the exact number of hospitalisations registered in the hospital information system (SIHSUS) from 2015. We applied the same proportion to the chronic cases of cases being monitored at the Schistosomiasis outpatient clinic (AE/HC/UFPE) that reported hospitalisation in the last 12 months for the scenarios of sensitivity analysis. The applied value was obtained through the average amount of a hospitalisation due to schistosomiasis reported in the SIHSUS.
	Hospitalisations in intensive care unit (ICU)	The number of hospitalisations in the ICU for Base Case was exactly the number of hospitalisations in ICU registered in SIHSUS/2015. For the scenarios of the analysis, we applied the proportion of hospitalised cases in ICU among the registrations from SIHSUS/2015. The applied value was the average value of a hospitalisation by schistosomiasis in an ICU bed in SIHSUS/2015.
Indirect health care costs	Transportation for outpatient follow-up	Mean value spent with transportation by patients monitored in the outpatient clinic (AE/HC/UFPE), applied to the local protocol of two medical appointments on average, per year, for each chronic patient. For the Base Case and the sensitivity scenarios, we applied the proportion of cases being monitored in the outpatient clinic that bore the costs of transportation for the follow-up.
	Caregiver’s payment	Mean value spent with caregiver by patients followed-up in the outpatient clinic (AE/HC/PE). For the Base Case and sensitivity scenarios, we applied the proportion of cases that paid a caregiver among patients being followed-up at the outpatient clinic (AE/HC/PE).
Indirect costs	Loss of wages due to premature death	Annual wage in 2015 (US$: 7,571.76), based on the average wage of US$: 567.88, including social and labor charges. The annual amount was applied to the estimated years of life lost (YLL) due to premature death in the economically active age range (15 to 65 years-old). The YLL number was kept constant in the Base case and sensitivity scenarios.
	Loss of wages due to hospitalisation and leave	Number of days lost due to hospitalisation and medical leave was 180 days for neurological cases and 90 days for other hospitalised cases. There was a 77.6% rate of economically active cases, which was the proportion of cases being followed at the outpatient clinic with formal employment.
	Disease aid	Benefit for 22.4% of the chronic cases in the Base Case and in sensitivity scenarios. We considered the proportion of cases being followed at the outpatient clinic and receiving disease aid due to schistosomiasis. The benefit value was calculated using the 2015 rule for workers receiving one minimum wage with 5 years of contribution.

Out of the 406 deaths (Table IV), 172 were in economical active ages (15 to 65 years). We calculated 5,187 YLL in these age groups with men contributing 2,903 (55.96%) of the YLL.

**TABLE III:** Number of deaths, mortality rate (MR) specific for schistosomiasis. Brazil, 2015

Federated state	Deaths	Population	Gross MR^*^	Standardised MR
Alagoas	54	3,340,528	1.62	1.48
Pernambuco	119	9,345,638	1.27	1.20
Sergipe	15	2,242,948	0.67	0.61
Rio Grande do Norte	4	3,442,158	0.12	0.46
Bahia	54	15,203.851	0.35	0.32
Minas Gerais	70	20,869,033	0.33	0.30
Paraíba	9	3,972,175	0.23	0.22
Espírito Santo	8	3,929,925	0.20	0.17
Distrito Federal	4	2,914,830	0.14	0.12
São Paulo	52	44,396,460	0.12	0.10
Maranhão	6	6,904,298	0.09	0.08
Goiás	5	6,610,683	0.08	0.06
Rondônia	1	1,768,162	0.06	0.05
Rio de Janeiro	4	16,550,009	0.02	0.02
Paraná	1	11,163,023	0.01	0.01
Brazil	406	204,450,380	0.12	0.18

MR: mortality rate. *: direct method of standardisation with stratification of deaths and population aged younger than 60, equal or older than 60 years. Source: SIM/SVS/MS 2010 Brazilian demographic census and estimates elaborated by the Department of Health/SVS/CGIAE.

Among direct healthcare costs, we estimated that US$ 2,1 million related to the diagnosis of schistosomiasis and its main complications and US$ 147,513.24 to the treatment of hepatointestinal and neurological infection forms. Non-healthcare direct costs such as transportation and domestic care from the patients’ perspective were estimated to be US$ 1,838.38; and indirect costs with leave, disease aid, and premature death totalled US$ 39,457,036.56. In the Base Case scenario, considering only schistosomiasis records of current information systems, 94.61% of the cost of illness was attributed to indirect costs. The total cost of schistosomiasis was estimated to be US$ 41,706,337.35.

The sensitivity analysis of the costs was performed using three scenarios, all of which resulted in total costs that were higher than those estimated in the Base Case. In Scenario B, which considered the estimate of infected people according to the Schistosomiasis National Survey, the estimated total costs were 72.59% higher than those of the Base Case. We also observed that the disease’s direct costs were higher in all the scenarios, with increased proportion to the total cost of the disease. In Scenario B (prevalence estimated in the survey), the direct healthcare costs were more than 1,000% higher than in the Base Case. In Scenario A (95% CI lower limit of the prevalence estimated in the survey), with the lowest estimated prevalence, the disease cost was 11.55% higher than the costs estimated in the Base Case. Table V shows the cost breakdown, according to category and type of sensitivity analysis.

## DISCUSSION

This study estimated 0.18 deaths for every 100 thousand residents in Brazil in 2015; and the highest rates were in the endemic states of Alagoas, followed by Pernambuco and Sergipe. There were 5,187 years of life lost due to premature death in economically active ages, and the disease costs were estimated at US$ 41,706,337.35 with 90% of these costs being indirect costs.

**TABLE IV:** Years of life lost (YLL) by schistosomiasis according to gender and per 1,000 residents. Brazil, 2015

Age range	Male		Female		Total			
	Deaths	YLL	Deaths	YLL	Population	Deaths	YLL	YLL/1,000^*^
1-4	1	89.51	0	0.00	14,737.740	1	89.51	0.01
5-14	1	80.03	0	0.00	32,671.352	1	80.03	0.00
15-29	3	210.21	2	135.16	51,373.431	5	345.37	0.01
30-44	22	1,169.30	12	647.26	47,437.888	34	1,816.56	0.04
45-59	51	2,032.37	35	1,356.45	34,289.353	86	3,388.82	0.10
60-69	56	1,568.85	55	1,538.11	13,641.753	111	3,106.96	0.23
70-79	39	761.75	62	1,199.70	6,990.107	101	1,961.45	0.28
80+	33	291.74	34	287.82	3,309.025	67	579.56	0.18
Total	206	6,203.76	200	5,164.49	204,450.649	406	11,368.26	0.06

*: per 1,000 residents. Source: SIM/SVS/MS. Estimates elaborated by the Department of Health/SVS/CGIAE.

**TABLE V:** Schistosomiasis mansoni cost and sensitivity analysis. Brazil, 2015

Category of cost		Type of cost	U$/case	Base Case		Scenario A		Scenario B		Scenario C	
				N	U$	N	U$	N	U$	N	U$
Direct health care costs	Diagnosis	Stool sample investigation	3.17	654.321	2,073,453.61	449.791	1,425,326.06	2,024.061	6,413,972.03	3,639.222	11,532,195.98
		HE form	57.55	397	22,847.61	11.245	647,164.74	50.601	2,912,155.01	90.981	5,236,077.84
		Neurological form	76.83	48	3,687.89	3.149	241,941.25	14.168	1,088,543.55	25.474	1,957,196.38
	Treatment	Infection	0.66	26.499	17,541.48	449.791	297,747.03	2,024.061	1,339,862.63	3,639.222	2,409,046.74
		HE outpatient clinic follow-up	158.44	397	62,900.71	11.245	1,781,658.76	50.601	8,017,226.78	90.980	14,414,879.01
		Neurological treatment	376.18	48	18,056.71	3.149	1,184,595.32	14.168	5,329,738.47	25.475	9,583,221.88
		Hospital ward hospitalisation	163.65	275	45,003.75	921	150,721.66	4.145	678,329.31	7.453	1,219,683.56
		ICU hospitalisation	445.62	9	4,010.59	29	12,923.00	133	59,267.54	239	106,503.32
Non- health care direct		Transportation	18.05	100	1,805.09	3.238	58,449.03	14.573	263,055.38	26.202	472,968.99
		Caregiver	2.08	16	33.29	504	1,048.78	2.226	4,632.10	4.076	8,481.78
Indirect costs		Leave and disease aid HE	1,703.65	87	148,217.28	544	926,783.94	2.449	4,172,231.38	15.532	26,455,528.04
		Leave and disease aid neurological	3,407.29	10	34,072.94	153	521,315.97	686	2,337,403.62	4.349	14,818,321.18
		Indirect cost (year lost) due to death	7,571.76	5.187	39,274,746.33	5.187	39,274,746.33	5.187	39,274,746.33	5.187	39,274,746.33
Summary of costs		Direct costs US$ (%)		2,249,340.79 (5.39)		5,801,575.33 (12.47)		26,106,782.80 (36.31)		46,940,255.48 (36.82)	
		Indirect costs US$ (%)		39,457,036.56 (94,61)		40,722,846.24 (87.53)		45,784,381.33 (63.69)		80,554,111.95 (82.86)	
		Total US$ (%)		41,706,337.35 (100)		46,524,421.58 (100)		71,981,164.14 (100)		127,494,367.43 (100)	

HE: hepatosplenic; ICU: intensive care unit.

Studies point out a decreasing trend of mortality due to schistosomiasis in Brazil,^13^ ^,^ ^14^ which suggests an annual reduction of around 2.8% per year. This decrease may be deemed multi-factorial (not mainly due to specific disease control actions) considering that the country has also undergone changes in healthcare systems and in various social and economic aspects throughout the years. However, schistosomiasis mortality reported in the SIM may be underestimated. There is a possibility that schistosomiasis deaths are being hidden by other causes in endemic states like Alagoas, Pernambuco, Sergipe, Bahia, and Minas Gerais. An investigation of active searches conducted in the largest emergency service of Pernambuco found that 30% of the digestive haemorrhage cases assisted in the unit was due to schistosomiasis.^15^

The chronic character of the disease should be considered when analysing its impact on mortality. Despite its potential severity, early diagnosis of the infection, even of the chronic forms, when followed by proper treatment, prevents the potentially lethal complications of the disease, especially haemorrhagic manifestations.^16^ Mortality caused by a disease that is closely related to poverty is not homogeneous and should be noticed as a sentinel indicator for monitoring the disease situation in Brazil.^17^ The states of Alagoas, Pernambuco and Sergipe exhibited the highest mortality rates. Even though Alagoas and Pernambuco showed the highest rates, the state of Sergipe may present a more serious concern for disease control because aside from its high mortality rate, it had the highest prevalence of the disease in the country based on the national survey.

The years of life lost due to premature death has been one of the indicators used in health loss estimates considering it is part of the disability-adjusted life year (DALY) indicator. The 2015 Global Burden of Disease (GBD) study estimated 382,543.93 years of life lost in the world from schistosomiases (with around 18,012 YLL in Brazil)^18^ and included almost 200 more deaths as compared to our study. Additionally, estimates of YLL produced by GBD show that Brazil had more YLL attributable to schistosomiasis as compared to Nigeria, Indonesia, China, and many other low-income countries.^17^ According to GBD, Brazil presented 228,101.29 YLL caused by neglected tropical diseases and malaria in 2015, and the highest numbers of YLL were related to Chagas disease (114,313.53), leishmaniasis (37,896,42.701), and dengue (38,195.82). Schistosomiasis represented 7.89% of the YLL in this group, greater than diseases like cysticercosis (2.02%) and malaria (1.68%).^18^ An important aspect to be considered regarding the years of life lost due to schistosomiasis is its preventability. One may infer that its chronic character causes slow reduction of the indicators involved in mortality. However, YLL considers that life expectancy at the age of death is higher the lower the age is. We observed deaths in people under 30 years-old in the states of Sergipe, Pernambuco, Minas Gerais and Bahia. The same chronic character of the disease may imply that deaths by schistosomiasis in young people represent a serious negative indicator of public health. This early loss of life significantly impacted the estimated costs of the disease.

This study presented significant costs associated with schistosomiasis even though it is a disease that could be prevented through better life conditions and health education. This disease is diagnosable and treatable in its early stages when measurements of effective control are systematically applied. Restoring health is expensive and depends on several resources that may be limited or rare. Collective treatment strategies of schistosomiasis in endemic localities are known to be affordable, cost-effective,^19^ ^,^ ^20^ and to contribute to the decrease in the occurrence of the disease. Consistent with this finding, we have estimated treatment cost of US$ 3.83 for each case diagnosed in stool sample investigations. There are only a few studies on the cost of diseases in Brazil. The cost associated with schistosomiasis was higher than for visceral leishmaniasis in 2014^21^ and lower than for diabetes in the state of São Paulo; this is a disease with an estimated prevalence for the country of around 6.8% of the population older than 18.^22^ Despite the methodological differences in studies on the cost of diseases, the most important thing to consider is that resources for healthcare are competitively allocated; and in complex epidemiological contexts (with significant prevalence of non-communicable diseases), diseases like schistosomiasis may lose visibility and priority.

Even if we only consider diagnosis and treatment (without the other cost categories nor the disabilities or losses of quality of life involved in the schistosomiasis burden), control actions in endemic places are expected to be more cost-effective than the management of more severe forms of the disease. Therefore, the performance of the healthcare system in controlling schistosomiasis is of paramount importance. Control actions based on stool sample diagnosis methods and treatment strategies based on positivity percentages are affected by the decrease of sensitivity of such methods in low-endemic areas.^23^ In addition, other researchers suggest that mass treatments in endemic places showed more effectiveness to control schistosomiasis transmission.^24^ If the treatment strategy in high prevalence areas were used, the costs associated with diagnosis and treatment of the infection as estimated in our study would be even lower. In Brazil, the treatment strategy is based on the outcomes of regular stool sample investigations. In the event of positivity lower than 15%, the people who tested positive would be treated; between 15% and 25%, those people living in the same household as the infected people would be treated as well; and above 25%, all people living in the entire area would be treated.^6^ The effectiveness of this strategy still needs to be assessed based on the costs associated with the disease and on the Kato-Katz sensitivity in low-prevalence areas.

The economic impact of schistosomiasis on both the healthcare system and the people affected by the disease is high. Elimination of schistosomiasis as a public health problem involves more than actively finding these cases and treating affected individuals. A study conducted in Kenya that monitored the impact of control programs based on collective treatment did not show evidence of transmission reduction during the program’s implementation even though it improved the health of individuals.^25^ This reinforces the urgent need of improving people’s life conditions, especially basic environmental sanitation, and people’s empowerment through health education. Brazil needs to improve its basic sanitation. In 2015, according to the Report of Public Expenses with Basic Sanitation, sewage network was available only to 59% of the population and such coverage is not homogeneous.^26^ In the Northeast, where endemic areas for schistosomiasis are found, this access is available for 49% of the cities in the urban areas and only for 3.84% in the rural areas. The urban areas of the Northeast have 6.7 million houses without access to the collector network. The distribution of such access according to income demonstrates inequality considering that the problem is mainly of the low-income population. In 2001 and 2015, the percentages of houses without access to the sewage collector network were 63% among households with the lowest income and 66% for those with income up to three times the minimum wages.^26^

Even with the current efforts to control schistosomiasis, its profile is still highly related to the conditions of life, population vulnerability, and permanent poverty.^4^ In endemic areas, contracting schistosomiasis does not generally occur in isolation. Its occurrence is concurrent with other intestinal parasites, other infectious diseases^4^ or even non-communicable diseases. The impact to economic and health conditions and quality of life related to this complex epidemiological profile still needs to be measured to develop integrated and effective interventions that would improve health and address inequality.^4^

There are limitations in our study, some of which are associated with the impact of the number of cases used in the accuracy of cost estimation and others are related to methodological options. The exclusive use of case records in information systems has probably underestimated the cost of schistosomiasis. Considering the differences between the clinical forms of the disease, which have a direct impact on costs, we needed to stratify the cases. Only SINAN presents the clinical form. Among more than 20 thousand positive cases registered in SISPCE, there may be cases with more advanced stages of the disease or with non-specific symptoms requiring other treatments (not only the specific pharmacological treatment), which would make the direct healthcare costs higher than our estimates. Additionally, the sensitivity analysis also pointed out more significant direct costs in the scenarios even when we used the lower limit of the confidence interval of the national survey estimates.

By presenting a study on the cost burden of schistosomiasis mansoni, our main intention is to show the economic burden that such a disease could impose on the entire society. These costs could represent potential benefits if schistosomiasis were eliminated. In our study, there were huge indirect costs related to the ‘market value’ of future contributions by people affected by the disease (had they been treated and thus, continued to work). Although there are some criticisms to this method, alternatives like the willingness to pay method (which may result in higher values than the human capital method), are difficult to use in studies on the cost burden of diseases.^12^

*In conclusion* - The economic burden of schistosomiasis mansoni disease in Brazil is high, and its greater impact is related to the loss of productivity. This disease is exacerbated by poor living and health conditions in endemic areas. Healthcare initiatives designed to eliminate its transmission will not be sustainable without basic sanitation, access to intra-domiciliary potable water, proper shelter, education, and access to healthcare. The persistence of this disease in Brazil is a challenge not only to public health but also to various sectors, especially to the political class that has the means to influence the mobilisation of public resources.
