# Supplementary material for: The cost of a disease targeted for elimination in Brazil: the case of schistosomiasis mansoni
Source: Mem Inst Oswaldo Cruz. 2019 Jan 14;114:e180347. doi: 10.1590/0074-02760180347 (PMC6340134; doi:10.1590/0074-02760180347)
Supplement: Supplementary file 1 [file 1678-8060-mioc-114-e180347-s.pdf]

TABLE

Costing method detail to estimate the schistosomiasis mansoni cost in Brazil, 2015

<table>
<tr><th>Category of cost</th><th>Category of cases</th><th>Items</th><th>Hypotheses</th><th>Source of values</th><th>Data source and references for the development of hypotheses</th></tr>
<tr><td rowspan="7">Health care direct costs</td><td rowspan="7">Stool sample investigations</td><td>Specimen container</td><td>Specimen container for each infected individual that received the item to perform the Kato-katz exam in the stool sample investigation. 1% waste.</td><td>Health price database - Averageprices 2015.</td><td rowspan="7">• Health Surveillance. Vigilância da esquistossomose mansoni: diretrizes técnicas 4. ed.- Brasília: Ministério da Saúde; Procedimentos operacionais padrão Laboratório Central de Saúde Pública.
• Information of inputs contained in the topic ‘Description in virtual stores, national scope of several brands to estimate the input weight’. The disposal amount was calculated based on the technical description of the Helmin Test Guide. (qualitative-quantitative test for parasitological detection of worm infections according to the Kato-katz method).
• Decree n. 2,488 from October 21st of 2011. Ministry of Health.
• Interview with key professionals in the laboratory area of public health, clinical management of schistosomiasis cases.</td></tr>
<tr><td>Kato-katz</td><td>One unit of the test for each subject who performed the technique in the stool sample investigations.</td><td>Value reported by the National Program on Schistosomiasis Control</td></tr>
<tr><td>Procedure gloves</td><td>One pair for each test performed in the stool sample investigations. 1% waste.</td><td>Health price database - Averageprices 2015.</td></tr>
<tr><td>Smear</td><td>A microscopy thick smear for every 10 exams performed in the stool sample investigation is washed and reused, without considering the cost of reprocessing a smear. 10% waste.</td><td>Health price database - Averageprices 2015.</td></tr>
<tr><td>Disposal</td><td>Disposal of gloves and smears estimated per kilo of waste produced in stool sample investigations. For the gloves, the weight per unit of 6.25g was considered. For smears, the unit weight of 0.5g was considered by adjusting a smear for every 10 exams. Total waste per exam = 12.55g of waste per exam at the cost of = US$: 0.00052, based on the investigated cost for 1kg of garbage (US$: 0.043)</td><td>Values regarding the contracts of urban garbage from the city of Recife in the year of 2015.</td></tr>
<tr><td>Field form - Stool sample exam and treatment</td><td>Printed sheet of the form for every 15 people approached in the stool sample investigation. Form used for registration of the result, later typing, and definition of the treatment strategy. 1% waste.</td><td>Average price of large-scale copy in 2015.</td></tr>
<tr><td>Technician to type the test</td><td>Wage value of a lab technician with complete high school, attributed to every exam, based on the average wage of lab technician in 2015 estimated in US$ 644.19. To define the value, we estimated the hour value (US$ 2.68) and work value of the technician for each performed test = US$ 0.89. We considered the time of 20 minutes per Kato-Katz exam (smear preparation, two readings, and processing of the smear for reuse).</td><td>Wage survey Datafolha 2015.</td></tr>
</table>

CC BY

		Health Agent	Monthly wage of an agent (US$: 310.75) weighted for providing service to 350 people (US$: 0.88) considering two visitations (US$ 1.77), in which one is for specimen collector distribution and mobilization and the other is to deliver results and treatment, when necessary).	ACS 2015 Wage average. Law N. 12.944 from June 17th, 2014.	
	Infection treatment	Praziquantel	Consumption of six tablets per positive case	National Program of Schistosomiasis Surveillance and Control	
		Digestive ultrasonography	Consumption of one exam per hepatosplenic case	SIGTAP	
		EDA	Consumption of one exam per hepatosplenic case		
		Rectal anatomopathological	Consumption of one exam for 50% of the hepatosplenic cases.		
		Stool sample test	One stool sample test for each estimated hepatosplenic case		
		Serology	One exam for 80% of the estimated hepatosplenic cases.		
		US/YEAR	Consumption of one exam per hepatosplenic case		
		EDA / year	Consumption of one exam per hepatosplenic case		
		Notification and investigation form for detected severe cases	Consumption of a printed sheet of the Notification and investigation form for every case reported in the National SINAN. 10% waste.	Market price, internet search	
	Outpatient treatment of hepatosplenic forms	Praziquantel	Consumption of six tables for every estimated case of the hepatosplenic form	National Program of Schistosomiasis Surveillance and Control	
		Treatment of liver diseases	Consumption of one treatment for every estimated case of the hepatosplenic form	SIGTAP	
		Total proteins and fractions	Consumption of two exams/year by technique for each estimated case of the hepatosplenic form		
		Complete blood count			
		Fasting blood glucose			
		Urea			
		Creatinine			
		TGO			
		TGP			
		Alkaline phosphatases			
		Total cholesterol			
		LDL Cholesterol			
		HDL Cholesterol			
		Triglycerides			
		Medication of regular use (omeprazole)	Consumption of two tablets per day/continuously (365 days) for every estimated case of the hepatosplenic form	Health Prices Database	

	Hospitalisation	Average cost of a hospitalisation (from admission to discharge) in hospital ward bed	Average value of a hospitalisation due to schistosomiasis in SIH-SUS, regardless of the duration.	Average value of one hospitalisation based on the SIHSUS/2015 database	Hospital information system (SIH-SUS)
		Average cost of a hospitalisation (from admission to discharge) in ICU	Average value of a hospitalisation due to schistosomiasis in SIH-SUS in ICU bed, regardless of duration.	Average value of one hospitalisation in ICU based on the 2015 SIH database	
Non health care direct	Transportation	Transportation for annual outpatient follow-up	Average value spent with transportation by patients adjusted for two appointments per year (US$ 9.02*2 = US$ 18.04). Cost per case of the hepatosplenic/neurological form per year	Primary data collected in the schistosomiasis outpatient clinic at Hospital das Clínicas from Universidade Federal de Pernambuco.	
	Caregiver	Caregiver's payment	Average value spent with caregiver (US$ 61.29) for 3.4% (5) of the estimated cases of chronic forms		
	Diagnosis of the neurological forms	Serology	One test for 80% of the estimated cases	SIGTAP	Guide of epidemiological surveillance and control of Schistosomal myeloradiculopathy Ministry of Health, 2006.
		LCR	Consumption of one exam for estimated cases of neuroschistosomiasis		
		LCR culture	Consumption of one exam for estimated cases of neuroschistosomiasis		
		Notification and investigation form for detected severe cases	Consumption of a printed sheet of the Notification and investigation form for every case reported in the National SINAN. 10% waste.		
		EAS	One exam for every technique per neurological form case		
		Urine drop gram			
		Uroculture			
		B12 vitamin			
		Anti-cardiolipin antibodies			
		Chest radiography			
		FAN			
		Anti HIV			
		Anti HTLV			
		VDRL			
		FTA-ABS (Igg and IGM)			
		HBs-Ag			
		Total anti Hbc			
		Anti HCV			
		Lupus anticoagulant		Private Network Laboratories (average)	
	Treatment of the neurological forms	Ivermectin, 200 micrograms/kg of weight	One treatment for every estimated case of neurological form	Health Prices Database	
		Ranitidine	1 350mg tablet per day for 180 days for the estimated cases of neurological form		
		Corticotherapy: prednisone, 1mg/kg of weight, single dose VO, in the morning, for six months	60mg dose of prednisone per day, per patient, for 180 days for each estimated cases of neurological form		
		Pulse therapy with methyl prednisolone, 15 mg/kg of weight/day, maximum dose of 1g, EV for five days	Methyl prednisone 1g day for 5 days for each estimated case of neurological form		

		Myelitis treatment	One treatment for every estimated case of neurological form	SIGTAP	
		Motor physical therapy	Two sessions per week for six months for the estimated cases of neurological form = 52 sessions of neurological rehabilitation		
		Psychotherapy and occupational therapy	A session of psychotherapy or occupational therapy per week for six months, for the estimated cases of neurological form = 26 sessions		
		Praziquantel	Consumption of six tablets for every estimated case	National Program of Schistosomiasis Surveillance and Control	
Non health care direct	Transportation	Transportation	Transportation for annual outpatient follow-up Average value spent with transportation by patients in Pernambuco (4) adjusted for two appointments per year (US$ 9.02*2 = US$ 18.05). Cost per case of hepatosplenic/neurological form per year	Primary data collected in the schistosomiasis outpatient clinic at Hospital das Clínicas from Universidade Federal de Pernambuco.	Primary data collected in the schistosomiasis outpatient clinic at Hospital das Clínicas from Universidade Federal de Pernambuco.
	Caregiver	Caregiver	Caregiver’s payment. Average value spent with caregiver (US$ 61.29) for 3.4% (5) of the estimated cases of chronic forms		
		Minimum wage, or lost or leave	Six months of leave were estimated for neurological cases, adjusted for the annual value per neurological case	Ministry of Planning. 2015	Guide of epidemiological surveillance and control of Schistosomal myeloradiculopathy Ministry of Health, 2006.
			Estimation of three months of leave for hepatosplenic cases, adjusted for the annual value per hepatosplenic case		Vigilância em Saúde. Vigilância da esquistossomose mansoni: diretrizes técnicas 4. ed.– Brasília: Ministério da Saúde
		Disease aid	We estimated the disease aid. For 22.4% (6) of the chronic cases estimated for 12 months, in the value of US$ 255.62 calculated through the 2015 rule	Rule for disease aid Law 13,135/2015	Primary data collected in the schistosomiasis outpatient clinic at Hospital das Clínicas from Universidade Federal de Pernambuco.
	Deaths	Loss of wages for the society by premature death	Estimated years of life lost. Annual mean wage from 2015 in the employer's perspective	Primary data collected in the schistosomiasis outpatient clinic at Hospital das Clínicas from Universidade Federal de Pernambuco.	Mortality information system.
